# Acute Hepatitis E Infection Associated With Deer Meat in the United States

**DOI:** 10.14309/crj.0000000000001068

**Published:** 2023-06-28

**Authors:** Kesiena Akpoigbe, Joan Culpepper-Morgan, Okeoghene Akpoigbe, Peter Santogade

**Affiliations:** 1 Division of Gastroenterology, Columbia University Medical Center, Health + Hospitals/Harlem Affiliation, New York, NY

**Keywords:** acute hepatitis, hepatitis E, deer, zoonosis

## Abstract

Acute hepatitis E virus (HEV) infection in the United States of America (U.S.A) is low. However, seroprevalence rate is about of 6%. Most cases of HEV infection have been reported from travelers from endemic countries with poor sanitary conditions. Evidence of HEV as a zoonotic infection has been reported from developed countries from swine and wild animals including boar and deer. There is no reported case of direct transmission from wild game to humans in the U.S.A. We report a case of HEV from butchering of deer meat.

## INTRODUCTION

Few cases of acute hepatitis E virus (HEV) infection have been reported in the United States; however, the seroprevalence rate is very high with one estimate of 6%–21%.^1–3^ Most reported cases of HEV infection are from travelers from endemic countries.^4,5^ Locally, reported cases of acquired HEV have been suspected from the consumption of pork harboring the virus suggesting a food-borne or zoonotic infection.^5–7^ HEV has also been detected in wild boar and deer in developed countries.^4,8,9^ Although the seroprevalence of HEV among deer in Canada ranges from 3.2% to 8.8%, the HEV viral RNA itself has not been isolated from the animals.^10^ But, circulation virus has been found in wild boar and deer in other developed countries.^8,11^ There is a documented zoonotic transmission of HEV infection from deer meat consumption in Japan and Canada.^4,9,12^ However, there has been no reported case of direct transmission between human and deer in the United States. We report a rare case of HEV infection after butchering of several deer in Wisconsin, USA.

## CASE REPORT

A 53-year-old Native American woman living on a reservation presented to a hospital in Wisconsin complaining of 1-week worsening of epigastric pain, nausea, vomiting, fatigue, and intermittent fever. She is a current daily smoker (10 cigarettes/d) and has 1-2 alcoholic drinks once or twice per month. No known allergy was found. Her medications include aspirin, citalopram, atorvastatin 20 mg, and melatonin. She has mild sclera icterus. Her abdomen was soft with epigastric tenderness. No hepatomegaly, spider nevi, or caput medusa was present. No clinical features concerning for hepatic encephalopathy were present. Her liver enzymes and bilirubin were significantly elevated, and a working diagnosis of acute hepatitis was established. Hepatitis B, C, and A tests were negative. Further workup to unravel the cause of her acute hepatitis included antismooth muscle antibody, Liver-Kidney-Microsomal-1 antibody, antimitochondria antibody, and hemochromatosis, which were all negative. A right upper-quadrant ultrasound imaging of the liver did not show any vascular, biliary, or pancreatic abnormalities. On further testing for hepatitis E, IgM and IgG were both positive as shown in Table 1. Since the patient gave a history of butchering 5 deer over 3 weeks ago, a high index of zoonotic transmission was thus suspected, and an assessment of acute HEV infection was made based on the serological test and elevated liver enzymes.

**Table 1.** Chronological description of events during hospitilization

Time	January 15, 2021	January 16, 2021	January 19, 2021	January 21, 2021	January 15, 2021
	Day 0	Day 1	Day 4	Day 6	Day 27
ALT	2,835	2,365	1,149	—	39
AST	1,546	1,107	198	—	22
ALK	262	243	211	—	104
Total bilirubin	2.6	1.8	0.6	—	0.3
Albumin	3.1	2.9	3.2	—	3.8
INR	1.0	—	—	—	—
Anti-HEV	IgG+	IgG+	IgG+	IgM/G+/+	IgM/G+/+

ALK, alkaline phosphatase; ALT, alanine aminotransaminase; AST, aspartate transaminase; HEV, hepatitis E virus; INR, international normalize ratio.

She was hospitalized for 4 days and managed conservatively with intravenous fluids and analgesics without antiviral therapy. Her liver enzymes and bilirubin from day 0 continued to trend down and returned to normal level when she was seen in the clinic after a month without any symptoms as shown in Table 1 below.

## DISCUSSION

Geographically, Wisconsin is very close to Canada, and deer crisscross between them. Unlike in the United States, HEV seroprevalence among deer in Canada is well documented with 8.8% in white-tailed deer, 4.5% in mule deer, and 3.2% in caribou.^10^ Although no active HEV RNA has been found in these animals from Canada, constant HEV circulation is well known in deer from studies in developed countries and is a source of concern for zoonotic infection and provides evidence of circulating HEV in deer.^8,11^ Cases have been reported from families infected with HEV virus from eating uncooked Sika deer meat like sushi or sashimi in Japan and Caribou in Canada.^4,9,12^ These animals are an important component of diet in these communities. Also, there is a high risk of exposure of HEV infection in humans with exposure to infected animals especially for slaughterers.^13^ This patient butchered deer and did not consume it. Butchering of the infected animal and fecal-oral transmission was the likely source of HEV.

Most HEV infections in the United States are asymptomatic, but <5% will develop acute symptomatic infection, which our patient did. But, it can progress to chronic hepatitis in immunocompromised individuals.^14^ The overall mortality of HEV is low <1% and most often higher in pregnant women where mortality could be as high as 25%.^7,15^ Infections are usually acute with self-limiting disease characterized by abrupt elevation in liver enzymes and bilirubin, and resolution of clinical and laboratory manifestations is in a few weeks. This was in keeping with the presentation of our patient, which showed elevated liver enzymes that trended down over a few weeks with resolution of symptoms, despite been positive for both IgM and IgG a month later.

One challenge with the report is the missing genotype of the HEV. Several genotypes of HEV have been identified.^16^ HEV-3 is known to circulate between humans and animals including deer. We may not know for sure which genotype our patient presented. This was not analyzed both in our subject or assessed in the deer that were butchered. Further analysis will be prudent to give credence to this and source confirming zoonotic infection.

The above case highlights the rare transmission of HEV between deer and humans in the United States. This finding provides temporal evidence of HEV as a zoonotic infection. This was similar to the cases reported from Japan from the consumption of undercooked deer meat. Attention to HEV as a zoonotic source and endemic in developed countries is needed.

## DISCLOSURES

Author contributions: K. Akpoigbe is the article guarantor. All authors were involved in the drafting and editing of this manuscript.

Financial disclosure: None to report.

Previous presentation: Presented at ACG 2022 Annual Meeting; October 25, 2022; Charlotte, NC.

Informed consent was obtained for this case report.
